# Transient Global Amnesia Linked to Impairment of Brain Venous Drainage: An Ultrasound Investigation

**DOI:** 10.3389/fneur.2019.00067

**Published:** 2019-02-05

**Authors:** Ke Han, Han-Hwa Hu, A-Ching Chao, Feng-Chi Chang, Chih-Ping Chung, Hung-Yi Hsu, Wen-Yung Sheng, Jiang Wu

**Affiliations:** ^1^Department of Neurology, The Seventh Affiliated Hospital, Sun Yat-sen University, Shenzhen, China; ^2^Graduate Institute of Clinical Medicine, College of Medicine, Taipei Medical University, Taipei, Taiwan; Cerebrovascular Treatment and Research Center, College of Medicine, Taipei Medical University, Taipei, Taiwan; Department of Neurology, Taipei Medical University-Shaung Ho Hospital, Taipei, Taiwan; ^3^Department of Neurology, College of Medicine, Kaohsiung Medical University and Department of Neurology, Kaohsiung Medical University Hospital, Kaohsiung, Taiwan; ^4^Department of Radiology, Taipei Veterans General Hospital and National Yang Ming University, Taipei, Taiwan; ^5^Department of Neurology, Taipei Veterans General Hospital and National Yang-Ming University, Taipei, Taiwan; ^6^Department of Neurology, Tungs' Taichung Metro Harbor Hospital, Taichung, Taiwan; ^7^Department of Neurology, First Hospital of Jilin University, Changchun, China

**Keywords:** internal jugular vein (IJV), magnetic resonance venography (MRV), transient global amnesia (TGA), ultrasound, Valsalva maneuver (VM), vertebral vein (VV), hemodynamics

## Abstract

**Background:** Previous neuroimaging and ultrasound studies suggested that compression and stenosis of the internal jugular vein (IJV) in patients with transient global amnesia (TGA) may impair IJV drainage, while a patent IJV releases intracranial pressure caused by the Valsalva maneuver (VM).

**Methods:** Seventy-nine TGA patients with complete ultrasound examination data during admission were recruited prospectively to evaluate IJV drainage, which included the time-averaged mean velocity, and the cross-sectional lumen area of the IJV at the vein's middle (J2) and distal (J3) segments and the cross-sectional area during a 10-s VM to test for any retrograde or anti-grade flow. Forty-five TGA patients and 45 age- and sex-matched control subjects underwent complete contrast-enhanced magnetic resonance (MR) venous studies, which included time-resolved imaging of contrast kinetics, contrast-enhanced axial T1-weighted MR imaging, and phase-contrast-based non-contrast enhanced magnetic resonance venography (MRV).

**Results:** In those subjects with complete MRV studies, the flow volumes exhibited at both the J2 and J3 segments of the left IJV and left vertebral vein (VV) were significantly lower in the TGA patients than in the control subjects. Although there was no significant difference in the flow volume of right IJV, the total of bilateral IJV, and VV flow volumes was still significantly lower in the TGA patients. As compared with the control subjects, the TGA patients exhibited significantly higher prevalence of completely blocked right IJV drainage at the J3 segment during the VM, but non-significantly higher for the left IJV at the J3 segment and for the right IJV at the J2 segment.

**Conclusion:** Our results confirmed that the total venous flow decreases in the IJVs and VVs of the patients with TGA. This is consistent with the findings of previous MR imaging studies that have reported about compression and stenosis of the draining veins. We also found that IJV drainage is relatively compromised during the VM in the patients with TGA.

## Introduction

Transient global amnesia (TGA) is defined as a sudden and transient inability to acquire new information (1). It can be triggered by certain events including Valsalva maneuver (VM)–like activities (1–3). Cerebral venous congestion/hypertension is one of the conditions that has been linked to TGA, which results from venous reflux while performing a VM in the subjects with internal jugular vein valve incompetence (IJVVI) (4–6). However, previous ultrasound and non-contrast venous magnetic resonance (MR) angiography studies have not supported a causal relationship between IJVVI and TGA (7–9). Previous studies have also reported that VM-induced pressure in the chest and abdomen is mainly transmitted to the intracranium via the epidural venous plexus (10, 11). Theoretically, bilateral IJV patency is needed for the brain venous drainage, which is regarded as a protective mechanism against intracranial venous hypertension. Stenosis or obstruction of the IJV hinders the brain venous drainage. This can directly cause intracranial hypertension (12, 13) and impair the protective function of IJV during the VM, which worsens the increased intracranial pressure further (11, 14). Consistent with this hypothesis of the venous outflow obstruction, we have previously showed that many patients with TGA exhibit stenosis or obstruction of the left brachiocephalic vein (BCV) (15). Using MR imaging, we have also demonstrated that the patients with TGA manifest a higher prevalence of compression/stenosis of the bilateral IJVs and left BCV, and transverse sinus (TS) hypoplasia, which supports the hypothesized role of abnormal brain venous drainage in the pathogenesis of TGA (16). Hence, we hypothesized that the compression/stenosis of the bilateral IJVs and left BCV would impede the brain venous drainage, which would result in reduced IJV flow volumes exhibited in the patients with TGA. We further hypothesized that in the patients with TGA, the IJV drainage would be particularly blocked during the VM, resulting in a phenomenon known as “IJV non-patency.” This would impair the role of IJV drainage in releasing the intracranial pressure. We tested these hypotheses with ultrasound evaluations of the morphology and hemodynamics of the extracranial IJVs and vertebral veins (VVs) at rest and during the VM.

## Methods

### Study Design and Participants

The main study design and the participants have been described elsewhere (16). In brief, from January 2008 to December 2012, 79 patients with TGA were admitted to the Taipei Veterans General Hospital Neurology Department. All were examined by a neurologist, and TGA was diagnosed according to the criteria as modified and validated by Hodge and Warlow (1). All the TGA patients underwent complete ultrasound examination including analyses of the IJV responses during the VM; but of those, 34 patients had already underwent an emergency MRI at the emergency department to exclude the possibility of acute ischemic stroke, and hence did not undergo the complete MR venous study. The remaining 45 patients underwent complete ultrasound examinations and complete contrast-enhanced magnetic resonance (MR) venous studies, which included magnetic resonance imaging (MRI), magnetic resonance angiography (MRA), and magnetic resonance venography (MRV) assessments of the IJV drainage. We prospectively recruited 45 age- and sex-matched control subjects from the individuals who underwent physical check-ups and presented no history of neurologic signs or symptoms. However, we did not recruit any controls for the subgroup of those 34 patients who did not undergo complete venous MR imaging studies.

Those 45 control subjects also underwent complete MRI, MRA, and MRV studies and ultrasound assessments. In accordance with the regulations of our government and the regulations of the Ethics Committee of Taipei Veterans General Hospital and in compliance with the Declaration of Helsinki, all the participants provided informed written consent with their signatures. The study protocol was approved by the Taipei Veterans General Hospital's institutional review board, and the study was conducted according to the institutional guidelines. All the participants gave the written informed consent.

### Ultrasound Acquisition

**Figure 1:**
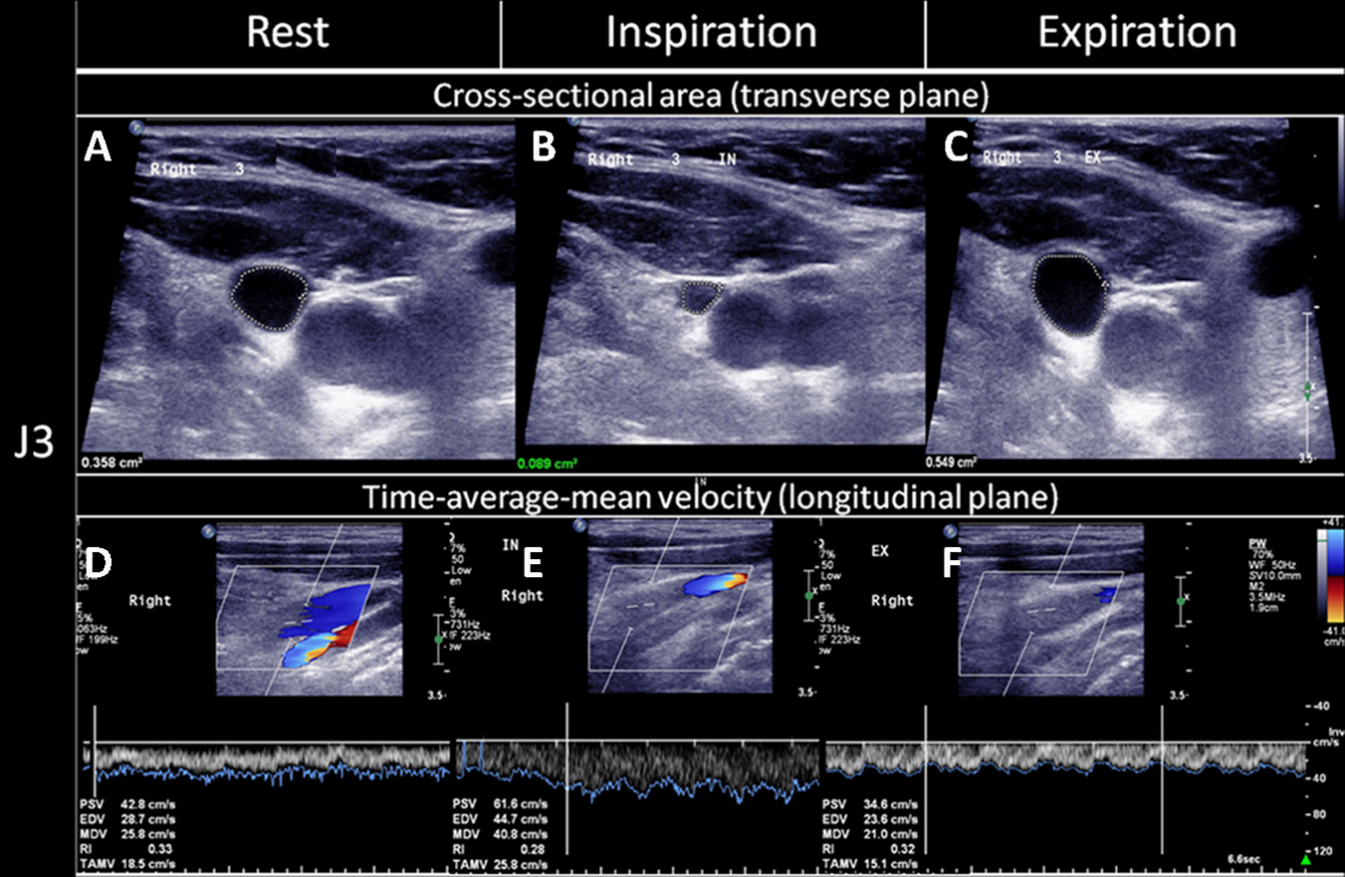
Quantitative evaluation of the CSAs and TAMVs of the IJVs during the three respiratory states. The cross-sectional lumen areas (CSAs) and time-average-mean velocities (TAMVs) of the upper segment of the internal jugular vein (IJV, J3) were recorded during a brief apnea after the three respiratory statuses, namely: **(A,D)** at rest (normal respiratory status, **(B,E)**; deep inspiration; and **(C,F)** expiration. As compared to that at rest, the CSA decreased **(B)** while the TAMV increased **(E)** during the deep inspiration, and the CSA increased **(C)** while the TAMV decreased **(F)** during the expiration. The figure was reproduced with the permission of Chao et al. (20).

**Figure 2:**
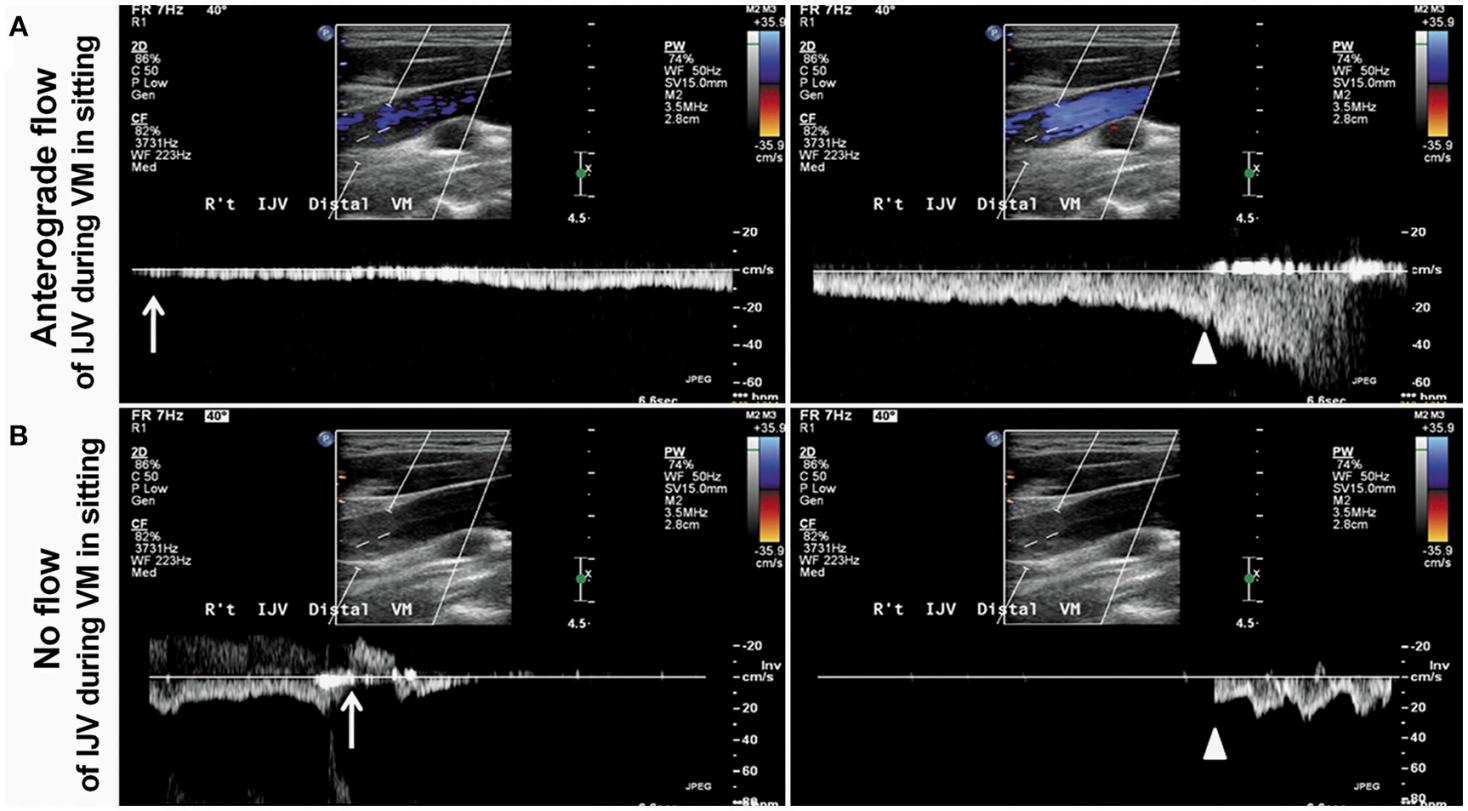
Display of Doppler spectra of the IJV (internal jugular vein) during the VM (Valsalva maneuver) in the sitting position. **(A)** Patient 1. An anterograde flow in the right IJV during the VM in the sitting position (upper **A**) suggests that the pressure can be released by reopening the IJV in a 42-year-old healthy female with patent IJV. **(B)** Patient 2. No flow in the right IJV during the VM in the sitting position (lower **B**) with a transient venous reflux (arrow, **B)** indicates that the intracranial venous pressure cannot be relieved effectively by the reopening the IJV in a 53-year-old TGA patient with significant IJV stenosis/obstruction. The arrows indicate the beginning of the VM, and the arrowheads indicate the end of the VM. The figure was reproduced with the permission of Han et al. (16).

All the participants underwent color-coded duplex sonography with a 7-MHz iU22 linear transducer (Philips Medical Systems, Andover, MA), performed within 7 days after their TGA attacks, by a technician who had more than 10 years of experience in venous ultrasound studies and was blinded to the subjects' characteristics. The method for ultrasound examinations of the extracranial venous system has been reported elsewhere (17–20). In brief, the IJV's time-averaged mean velocity (TAMV, cm/s) and the cross-sectional lumen area (CSA, cm^2^) were recorded at its middle (J2) and distal (J3) segments (19, 20). The location of J2 and J3 is the point where the common facial vein drains into the IJV (19, 20). The IJV segment above this point is J3, and below is J2. We acquired the TAMVs by directing the Doppler cursor parallel to the vein with the gate adjusted to comprise the entire lumen. We measured the TAMVs with the iU22's built-in software, and included at least three cardiac cycles on the Doppler spectrum. The probe was then turned by 90° at the same IJV segments to measure the CSAs, which were measured thrice as B-mode images. These images were averaged for later analysis. Both the CSAs and TAMVs were recorded at a brief apnea after the three respiratory states (20). The recordings made during the brief apnea after the three respiratory states are shown in the figures as follows: (1) normal respiration (resting or baseline) (Figures 1A,D), (2) deep inspiration (Figures 1B,E), and (3) expiration (Figures 1C,F). During the latter two respiratory states, the subjects were instructed to avoid strain-inducing breath-holding, because it could increase intra-thoracic pressure. We recorded the CSAs of the IJVs during a 10-s VM to test the incidence of reflow or no flow (Figure 2). The flow volume (FV) equals the TAMV multiplied by the CSA. Since it was difficult to obtain the CSAs of the VVs, we estimated the diameters of the VVs while modeling the VVs as having perfectly circular cross-sections. We measured the diameters of the VVs adjacent to the V2 segment of the vertebral artery. We also determined the jugular venous reflux (JVR) at the baseline and during the VM. Our methods for performing the VM and detecting the JVR have been described elsewhere (17, 21).

All the ultrasound data and color imaging records were collected prospectively. Two trained neurologists, who were also blinded to the subjects' clinical characteristics, reviewed the collected data for the CSAs, TAMVs, and FVs for the IJVs, and the diameters and TAMVs for the VVs. A consensus meeting was conducted to discuss any problems or disagreements.

### MR Imaging Study

Our methods for MRI and grading of TS hypoplasia and stenosis/compression of the IJV and BCV have been described elsewhere (16, 20). In brief, we performed contrast-enhanced MRI of all the participants using a 1.5-T Excite II MRI device (GE Medical Systems, Waukesha, WI), which included time-resolved imaging of contrast kinetics (TRICKS), contrast-enhanced axial T1-weighted MRI (Contrast T1), and phase-contrast based non-contrast enhanced MRV. All the patients were examined within 10 days after their TGA attacks.

The IJV morphologies were assessed at the level of upper IJV (C1–2 level) and middle IJV (C3–5 level) using Contrast T1. The IJV compression/stenosis was graded according to the following criteria (16, 22): grade 0 = normal round or ovoid appearance; grade 1 = mild flattening; grade 2 = moderate flattening; grade 3 = severe flattening or non-visualized appearance (Figure 1A).

Based on the filling defect shown on TRICKS, the left BCV obstruction was graded as follows: grade 0 = normal or compression ≤20%; grade 1 = compression >20% and ≤80%; grade 2 = compression >80%; grade 3 = grade 2 + presence of different types of venous collaterals (23).

Based on MRV and Contrast T1 studies, TS morphology was graded based on the criteria modified from that given by Han et al. (16) and Cheng et al. (24). All the asymmetrical percentages were calculated relative to the contralateral TS, as follows: grade 0 = symmetry or asymmetry ≤10%; grade 1 = asymmetry >10% and ≤50%; grade 2 = asymmetry >50%; grade 3 = aplasia or TS signal absent. Hypoplasia was defined as an asymmetry <50%. We have illustrated the locations for performing MRV and Contrast T1 examinations in our previous paper (16). However, in this study, only the findings of Contrast T1 were used for the analysis of hypoplasia.

All the MR imaging scans were examined by a neuroradiologist and a neurologist. The intra-class correlation coefficient for grading indicated an inter-rater reliability of 0.91. If inconsistencies resulted between the reports given by the two MRI readers, they discussed and reached a “consensus.”

### Statistical Analysis

Since this study was the first ultrasound study to evaluate the mechanism of jugular venous outflow impairment in the TGA patients, the sample size could not be pre-determined. The ultrasound data, such as the time-averaged mean velocity (TAMV), flow velocity (FV), and cross-sectional lumen (CSA) were skewed and were observed to be not normally distributed for the jugular venous flow. Thus, the expression of medians and inter-quartile ranges was used, and Wilcoxon rank sum analysis was performed. The chi-square test was performed for the categorical data. The same applies to the CSAs data for the TS, IJV, and VV. We compared the ultrasound data from the patients and control subjects using the Wilcoxon rank sum test. We used the chi-square tests to compare the prevalence of IJVVI or IJV patency during the VM. We defined the statistical significance as a 2-sided *p* < 0.05. All the analyses were performed with a SAS, 9.2 (SAS Institute, Cary, NC).

## Results

The demographic and clinical characteristics of all the 79 TGA patients, and the subset of 45 patients and 45 age- and sex-matched controls who underwent complete MRI, MRA, and MRV examinations are shown in Table 1. The patients and controls did not differ significantly in terms of their vascular risk factors. The TAMVs, FVs, and CSAs of the bilateral IJVs in the age- and sex-matched 45 patients and 45 controls are shown in Table 2. As compared to the controls, the TGA patients exhibited significantly lower TAMVS at the J2 and J3 segments of the bilateral IJVs. They also exhibited significantly lower FVs in the left IJV at the J2 and J3 segments and in the left VV. The FVs were not significantly lower in the right IJV; however, the patients still exhibited significantly lower total flow volumes in the bilateral IJVs and VVs. In both the patients and controls, the CSAs of the bilateral IJVs were not significantly different either at the J2 or J3 segment. The TGA patients exhibited a significantly higher prevalence of IJVVI (patients vs. controls: 82 vs. 44%); however, the side-specific prevalence was significantly greater on the left side (patients vs. controls: 53 vs. 20%), while the right-sided prevalence being comparable between the patients and controls (patients vs. controls: 29 vs. 24%). Furthermore, we detected a significant difference in the prevalence of left-sided IJVVI between the subjects with and without the presence of left BCV compression/stenosis [19 (61%) vs. 14 (24%), *p* = 0.0044].

**Table 1:** Demographic data and clinical features of TGA patients and controls.

	**TGA (total number of patients)**	**TGA patients with complete MRV study**	**Control subjects with complete MRV study**
	***n*** **=** **79**	***n*** **=** **45**	***n*** **=** **45**
**DEMOGRAPHIC**			
Age	61.4 ± 8.7(32–85)	61.5 ± 8.7(35–85)	61.5 ± 8.7(35–85)
Gender (M/F)	57/24	19/26	19/26
Coronary artery disease	2 (2.5%)	1 (2.2%)	0 (0%)
Hyperlipidemia	5 (6.3%)	3 (6.7%)	2 (4.4%)
Hypertension	8 (10.1%)	4 (8.9%)	5 (11.1%)
Diabetes mellitus (DM)	4 (5.0%)	2 (4.4%)	1 (2.2%)
Headache with cough	7 (8.8%)	4 (8.9%)	0 (0%)
Mitral valve prolapse (MVP)	6 (7.6%)	3 (6.7%)	2 (4.4%)
Sleep apnea syndrome (SAS)	2 (2.2%)	1 (2.2%)	0 (0%)
Syncope	5 (6.3%)	3 (6.7%)	0 (0%)
Insomnia	3 (3.8%)	1 (2.7%)	0 (0%)
Glaucoma	5 (6.3%)	2 (4.4%)	0 (0%)
Carotid stenosis	1 (1.3%)	0 (0%)	0 (0%)
MCA stenosis	5 (6.3%)	3 (6.7%)	0 (0%)
Previous stroke	3 (3.8%)	1 (2.2%)	0 (0%)
**CLINICAL PROFILES OF TGA**			
Recurrent	35 (44.3%)	21 (46.7%)	
Duration of amnesia (hours)	7.9 ± 8.3 (0.2–14)	7.5 ± 7.9 (0.2–11)	
VM–like activities or precipitating factors, *n* (%)	26 (32.9%)	16 (35.6%)	

*TGA, Transient Global Amnesia*.

**Table 2:** Comparisons of the TAMVs, FVs, and CSAs of the IJVs between the age- and sex-matched TGA patients and control subjects.

**Segment**	**Parameter**	**Patients with TGA** ***n=*** **45**		**Control subjects** ***n=*** **45**	
		**Left**	**Right**	**Left**	**Right**
J2	TAMV (mm/s)	72.4^**^ (45.6–109.0)	143.0^*^ (103.0–206.0)	98.9 (61.2–158.0)	152.0 (91.0–231.0)
	CSA (mm^2^)	60 (34–75)	73 (47–101)	45 (35–76)	75 (47–105)
	FV (mL/min)	3.53^*^ (2.54–6.38)	12.14 (6.81–15.86)	4.35 (3.30–8.72)	15.95 (11.43–22.34)
J3	TAMV (mm/s)	79.6^*^ (41.9–134.0)	147.0^*^ (97.0–203.0)	101.0 (67.4–164.0)	189.0 (141.0–208.0)
	CSA (mm^2^)	22 (17–30)	39 (24–66)	29 (15–37)	34 (23–43)
	FV (mL/min)	1.35^*^ (0.59–3.32)	5.68 (2.89–10.53)	3.09 (1.67–4.52)	6.61 (4.02–7.51)
IJV	Reflux (number of cases)	24 (53%)^**^	13 (29%)	9 (20%)	11 (24%)
VV	FV (mL/min)	2.03^**^ (0.76–4.33)	8.40 (3.40–14.04)	5.80 (1.65–23.27)	13.13 (3.87–16.70)
J2	Total FV (mL/min)	15.71 (12.31–20.59)		16.29 (13.55–22.98)	
J3	Total FV (mL/min)	9.68 (5.08–12.22)		9.40 (6.40–12.18)	
Bilateral J2 FVs + Bilateral VV FVs	Total FV(mL/min)	29.43^*^ (19.17–39.10)		36.17 (29.44–52.54)	

CSA, Cross-Section Lumen Area; FV, Flow Volume; IJV, Internal Jugular Vein; TAMV, Time-Averaged Mean Velocity; TGA, Transient Global Amnesia; VV, Vertebral Vein;

* p < 0.05: when comparing the TGA patients and controls (ipsilateral IJV);

** *p < 0.01: when comparing the TGA patients and controls (ipsilateral IJV)*.

We have often observed that the IJV drainage flow usually appears ~4–8 s after initiating the VM, so we simply defined the complete absence of IJV drainage flow at the J2 or J3 segment within 10 s of initiating the VM as IJV “non-patency.” Table 3 displays the relationship between the ultrasound findings of IJV non-patency during the VM, and the MRI findings of venous compression/obstruction or TS hypoplasia in those 90 study subjects (45 patients and 45 controls). For the left IJV, the prevalence of ultrasound-detected IJV non-patency during the VM was significantly greater at the J2 segment in the study subjects with an upstream TS hypoplasia than that in the patients without such hypoplasia (56.9 vs. 44.3%, respectively; *p* = 0.0425). For the right IJV, the prevalence of IJV non-patency at the J2 segment was significantly higher in the patients with IJV compression at C1 or C4 than in the patients without such compression (62.07 vs. 28.57%, respectively; *p* = 0.0111). We found no significant difference in the prevalence of bilateral IJV non-patency during the VM between those age- and sex-matched patients and controls who underwent complete MRI examinations. However, since there are no statistical differences in all the flow profiles between two groups of TGA patients with and without venous MR imaging as shown in Table S1, thus we included the 34 patients who underwent complete ultrasound examinations but incomplete MRI examinations for analysis, we found a significant difference in the prevalence of IJV non-patency during the VM between the patients and controls (Table 4). Specifically, we found that the patients exhibited significantly higher IJV non-patency at the right J3 segment (patients: 32.1%; controls: 11.6%; *p* = 0.0128), but not significantly higher in the left J3 segment (patients: 49.35%; controls: 37.21%), and the right J2 segment (patients: 44.00%; controls: 32.56%).

**Table 3:** The prevalence of no-reflow in the IJVs with and without venous compression/stenosis during the VM in the study subjects with complete MR venous examination.

**Venous compression/stenosis**	**Segment**	**No-reflow in the IJVs during the VM**
TS hypoplasia in contrast T1 (*n* = 20)	J3	50/48%
	J2	56.9/44.3%^*^
Left C1 or C4 compression (Yes/No) (Yes = 42; No = 48)	J3	54.2/45.5%
	J2	75.0/72.7%
Right C1 or C4 compression (Yes/No) (Yes = 40; No = 50)	J3	36.7/10.7%^**^
	J2	62.1/28.6%^#^
BCV compression (Yes/No) (Yes = 31; No = 59)	J3	51.7/46.4%
	J2	44.8/46.4%

MR, Magnetic Resonance; BCV, Brachiocephalic Vein; IJV, Internal Jugular Vein; TS, Transverse Sinus; VM, Valsalva Maneuver;

* p = 0.0425;

** p = 0.021;

# *p = 0.0111*.

**Table 4:** The prevalence of no-reflow in the IJVs during the VM in all the 79 TGA patients and 45 controls among the study subjects with complete MR venous examination.

**Side**	**Segment**	**Patients with TGA *n =* 79**	**Controls *n =* 45**
Left	J3	49.35% (*n =* 77)	37.21% (*n =* 43)
Left	J2	70.89% (*n =* 79)	67.44% (*n =* 43)
Right	J3	32.05%^*^ (*n =* 78)	11.63%^*^ (*n =* 43)
Right	J2	44.00% (*n =* 75)	32.56% (*n =* 43)

IJV, Internal Jugular Vein; TGA, Transient Global Amnesia; VM, Valsalva Maneuver; MR, Magnetic Resonance;

* *p = 0.0128*.

## Discussion

Our results confirmed our first hypothesis that the patients with TGA having IJV stenosis/compression at various segments would exhibit significantly lower total FVs in the bilateral IJVs and VVs resulting in important consequences than that exhibited by the control subjects. More importantly, our findings are consistent with our second hypothesis that the prevalence of right IJV non-patency at various segments during the VM would be significantly higher in the study subjects with IJV stenosis/compression at various segments; and therefore, would be higher in the TGA patients than that in the controls, which supports our novel hypothesis of venous pathogenesis involved in the TGA attacks (16). Specifically, an insufficient IJV patency prevents the release of increased intracranial pressure and venous congestion/hypertension in the basilar plexus and cavernous sinus caused by VM-like maneuvers. Moreover, venous stasis and occlusion may cause constriction of cerebral arterioles (16), which further compromises cerebral hemodynamics.

### Venous Flow Velocity and FV in IJVs

This study, our previous study (20), and other studies (22, 23) all revealed that the IJV or BCV compression and stenosis significantly reduce the IJV FVs. These result in the venous drainage being routed through less efficient alternative routes, such as the tortuous path through the spinovertebral venous plexus. This obstruction of the venous drainage may also induce changes in the arterial blood flow, such as arterial constriction, through the venoarterial reflex (25). Such changes may explain the observation in our previous studies (21, 24) that, the patients with transient monocular blindness without carotid stenosis exhibited increased downstream resistance of the retrobulbar arteries (i.e., ophthalmic artery, posterior ciliary artery, and central artery) in association with significantly increased prevalence of compression/stenosis in the bilateral IJVs (26). In this study, we did not measure the venous FV in the spinovertebral venous plexus, because it is undetectable by the ultrasound; therefore, we do not know the changes in the total venous drainage from the bilateral TSs, though the patients exhibited significantly lower total FV in the bilateral IJVs and VVs.

### Prevalence of IJV Non-patency During the VM

Our results supported our second hypothesis that the prevalence of IJV non-patency during the VM would be higher in the TGA patients than that in the controls. We observed a significant difference in the prevalence of right IJV non-patency (patients: 32.1%; controls: 11.6%; *p* = 0.0128) as compared to that for the left IJV non- patency (patients: 49.4%; controls: 37.2%). An abundance of evidence indicates that VM-induced pressure from the chest and abdomen is mainly transmitted to the intracranial compartment via the epidural venous plexus or vertebral venous plexus. Orthograde IJV outflows emerging shortly after the beginning of the VM, thus, serve as a mechanism for regulating the intracranial pressure and equalizing the pressure within the venous system (10, 11, 14, 16). Our findings indicate that the patients with TGA may experience defective intracranial pressure regulation during the VM-like movements. As described earlier, we restricted our definition of IJV non-patency to complete absence of the IJV drainage at the J3 or J2 segment during the first 10 s of the VM; but, this consideration regarding the IJV drainage might be unexhaustive. Several patients with partial or limited IJV drainage were excluded from this definition, which may explain why we observed a lower prevalence of IJV non-patency during the VM. Further research is needed to develop a more sensitive and specific definition of IJV non-patency during the VM.

### Incompetence of Jugular Venous Valves

Cerebral venous congestion/hypertension, which results from the venous reflux during the VM consequent to the IJVVI, is linked to TGA (4–6). However, previous ultrasound studies using either retrograde flow (27) or air bubbles (28) during the VM explained the involvement of only the proximal region of the IJV in the IJVVI, ignoring the rest of the IJV and the entire BCV, and possibly missing other important IJV/BCV abnormalities. Unsurprisingly, previous ultrasound and non-contrast MRA results have not supported a causal relationship between the IJVVI and TGA (7–9). Other than the IJVVI, we have previously described three ultrasound patterns of IJV abnormalities in the patients with TGA: (i) an isolated reverse flow in the left jugular vein branch (JB), (ii) a segmental reverse flow in the left distal IJV, and (iii) a continuous reverse flow in the left IJV and JB (29). All the three of these IJV patterns are suggestive of the venous outflow obstruction or compression/stenosis of the left BCV (29). Similar to other studies (4–6), even in this study, the overall prevalence of IJVVI was higher in the TGA patients than that in the controls (Table 2); but, the prevalence was higher in the left IJV only, and not in the right IJV. Furthermore, we found that the prevalence of IJVVI was significantly higher in the individuals with left BCV compression/stenosis. This suggests that the IJVVI might occur secondary to the BCV compression/stenosis on the left side. The fact that the IJVVI was more frequently observed on the right side, as reported in other studies (28), raises the question of whether the jugular venous valves are vulnerable in cases of IJV compression/stenosis due to pressure imbalances across the valves or whether the previous air bubble methodology overestimated the IJVVI prevalence (30). Further research is needed to address this question.

### Assessments of IJV Compression With Different Imaging Modalities

It is worth mentioning that despite the patients exhibiting reduced flow velocities and FVs in each of the segments of the bilateral IJVs, the diameters of their IJV segments were comparable to that of the controls (Table 2). Moreover, the patients' right IJVs were slightly wider than that of the controls. This may be partially explained by the Bernoulli's equation, which states that the pressure in the venous lumen is inversely proportional to the flow rate, so that the lumen diameter may be enlarged with flow stasis resulting from the venous stenosis/compression. As described earlier, the FV of the IJV decreases in cases of IJV or BCV stenosis/compression (20, 22, 23). However, discrepancies in the diagnosis of IJV compression/stenosis may be observed due to different types of venous examination (31). Catheter venography has been traditionally regarded as the gold standard for diagnosing venous disorders involving compression/stenosis, but it does not measure the flow velocity or FV; hence, it cannot prove or disprove the MRI or ultrasound findings that indicate decreased IJV FV resulting from compression/stenosis.

### Study Limitations

This study has several limitations, particularly regarding the use of ultrasound to study the IJV. First, there are no ideal, fixed locations for measuring the CSA and TAMV in the J2 and J3 segments; and these measurements may vary if the IJVs are non-uniform in diameter, which may occur in the cases where the IJV is affected by segmental dilatation, narrowing, or compression. However, the FV was calculated by multiplying the TAMV by the CSA, and it was theoretically correct according to the Bernoulli's equation. We measured the CSA and TAMV at the widest available lumen of the J2 and J3 segments to minimize the bias. This allowed us to detect the differences in both the FVs and TAMVs between the patients and controls. Second, it is unexhaustive to consider complete absence of IJV drainage during the VM while explaining IJV non-patency. Several of our subjects exhibited limited or intermittent flow, which suggests partial IJV blockage. However, we excluded these subjects from our definition of IJV non-patency, which may have caused us to underestimate the prevalence of IJV non-patency during the VM. Third, left BCV blockage may disappear during deep inspiration (15), which usually precedes the VM; hence, paradoxical reopening of the left BCV and left IJV occurs during the VM. This causes further underestimation of the prevalence of non-patency.

## Conclusion

Our results further confirmed that decrease in the total flow of the IJVs and VVs reflect impaired venous drainage in the patients with TGA, which is consistent with the findings of previous MRI studies that reported about the compression/stenosis of the bilateral IJVs and the left BCV. Second, our findings support the hypothesis that the compression/stenosis of the bilateral IJVs and left BCV may block the IJV drainage during the VM, which may prevent the release of intracranial hypertension caused by the VM. Further study is needed to obtain a more sensitive and specific definition of IJV non-patency during the VM.

## Author Contributions

H-HH and JW conceived and designed the experiments. KH, A-CC, F-CC, C-PC, and H-YH performed the experiments. KH, W-YS, and H-HH analyzed the data. A-CC, F-CC, C-PC, and H-YH contributed materials/analysis tools. KH and H-HH wrote the paper.

### Conflict of Interest Statement

The authors declare that the research was conducted in the absence of any commercial or financial relationships that could be construed as a potential conflict of interest.
