# Supplementary material for: Transient Global Amnesia Linked to Impairment of Brain Venous Drainage: An Ultrasound Investigation
Source: Front Neurol. 2019 Feb 5;10:67. doi: 10.3389/fneur.2019.00067 (PMC6370701; doi:10.3389/fneur.2019.00067)
Supplement: Supplementary file 1 [file Table_1.DOCX]

**Table S1 Comparison of the ultrasound parameters of venous flow between patients with and without venous MR imaging**

| **Segment** | **Parameter** | **Patients with MR**  **n=45** | |  | **Patients without MR**  **n=34** | |
| --- | --- | --- | --- | --- | --- | --- |
|  |  | **Left** | **Right** |  | **Left** | **Right** |
| **J2** | TAMV (mm/s) | 72.4  (45.6-109.0) | 143.0 (103.0-206.0) |  | 65.4  (27.9-80.3) | 126.4  (93.2-187.9) |
|  | CSA (mm2) | 60  (34-75) | 73  (47-101) |  | 59  (34-86) | 87  (56-128) |
|  | FV (mL/min) | 3.53  (2.54-6.38) | 12.14 (6.81-15.86) |  | 3.74  (1.13-5.65) | 8.99  (6.12-14.84) |
| **J3** | TAMV (mm/s) | 79.6  (41.9-134.0) | 147.0  (97.0-203.0) |  | 73.8  (46.5-146.0) | 152.0  (118.0-205.0) |
|  | CSA (mm2) | 22  (17-30) | 39  (24-66) |  | 22.7  (14-45) | 46  (33-61) |
|  | FV (mL/min) | 1.35  (0.59-3.32) | 5.68 (2.89-10.53) |  | 1.82  (0.78-3.67) | 7.11  (4.21-9.55) |
| **IJV** | Reflux  (number of cases) | 24(53%) | 13 (29%) |  | 17 (50%) | 11(32%) |
| **VV** | FV (mL/min) | 2.03  (0.76-4.33) | 8.40  (3.40-14.04) |  | 4.82  (1.06-7.96) | 6.24  (3.28-13.20) |
| **J2** | Total FV (mL/min) | 15.71  (12.31-20.59) | |  | 14.01  (9.80-19.10) | |
| **J3** | Total FV (mL/min) | 9.68  (5.08-12.22) | |  | 10.57  (8.05-12.82) | |
| **Bilateral J2 Fvs+ Bilateral VV Fvs** | Total FV(mL/min) | 29.43  (19.17-39.10) | |  | 25.35  (18.39-37.38) | |

*CSA, Cross-Section Lumen Area; FV, Flow Volume; IJV, Internal Jugular Vein; TAMV, Time-Averaged Mean Velocity; TGA, Transient Global Amnesia; VV, Vertebral Vein;*
